# Supplementary material for: Association of CD206 Protein Expression with Immune Infiltration and Prognosis in Patients with Triple-Negative Breast Cancer
Source: Cancers (Basel). 2022 Oct 3;14(19):4829. doi: 10.3390/cancers14194829 (PMC9564167; doi:10.3390/cancers14194829)
Supplement: Supplementary file 1 [file cancers-14-04829-s001.zip › cancers-1843554-supplementary Figure S6.pdf]

CD206 prognostic significance in tumors with TILs ≤ 5%

A) OS B) RFS

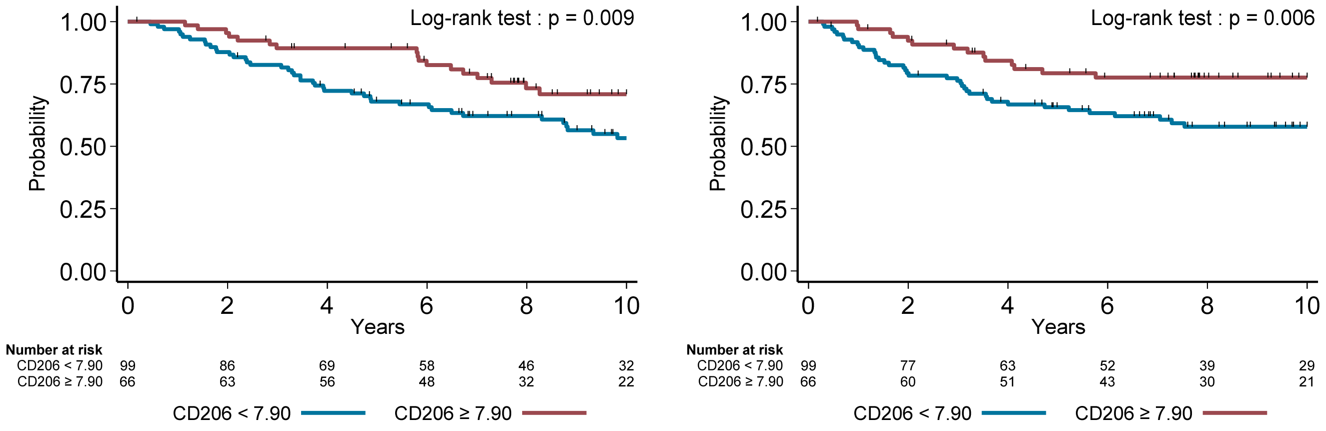

CD206 prognostic significance in tumors with TILs > 5%

C) OS D) RFS

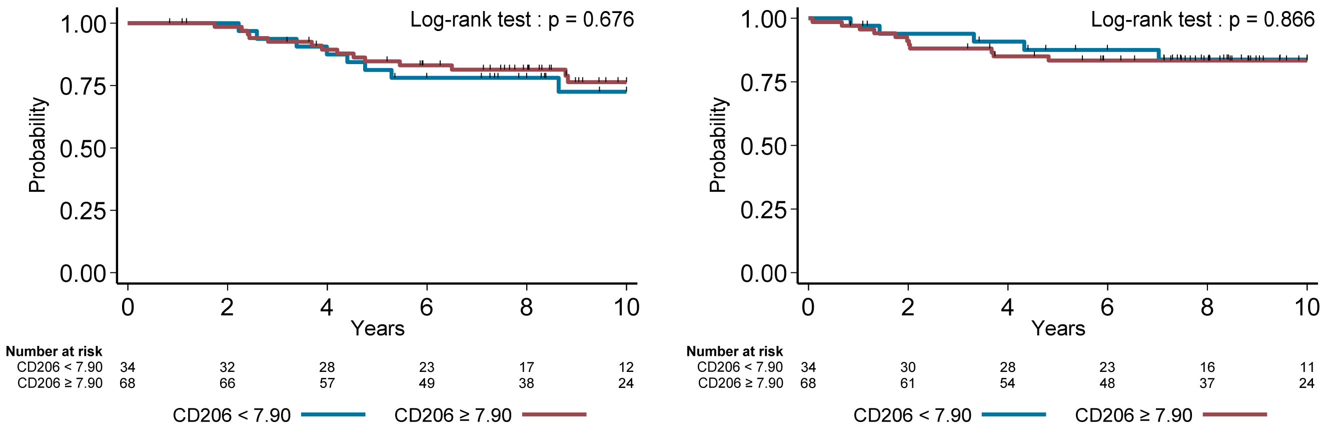

Supplementary Figure S6: **Influence of CD206 expression on overall survival (OS) (A and C) and relapse-free survival (RFS) (B and D) according to TILs level: TILs ≤5% (A and B) and TILs >5% (C and D).** Red lines represent high CD206 expression (≥7.90 cells/cm²) and blue lines represent low CD206 expression (<7.90 cells/mm²).
N=165 tumors with TILs ≤5%; N=102 tumors with TILs >5%.
