# Supplementary material for: Association of CD206 Protein Expression with Immune Infiltration and Prognosis in Patients with Triple-Negative Breast Cancer
Source: Cancers (Basel). 2022 Oct 3;14(19):4829. doi: 10.3390/cancers14194829 (PMC9564167; doi:10.3390/cancers14194829)
Supplement: Supplementary file 1 [file cancers-14-04829-s001.zip › Manuscript MacrophagesTNBC-Bobrie-SuppFigureS1.pdf]

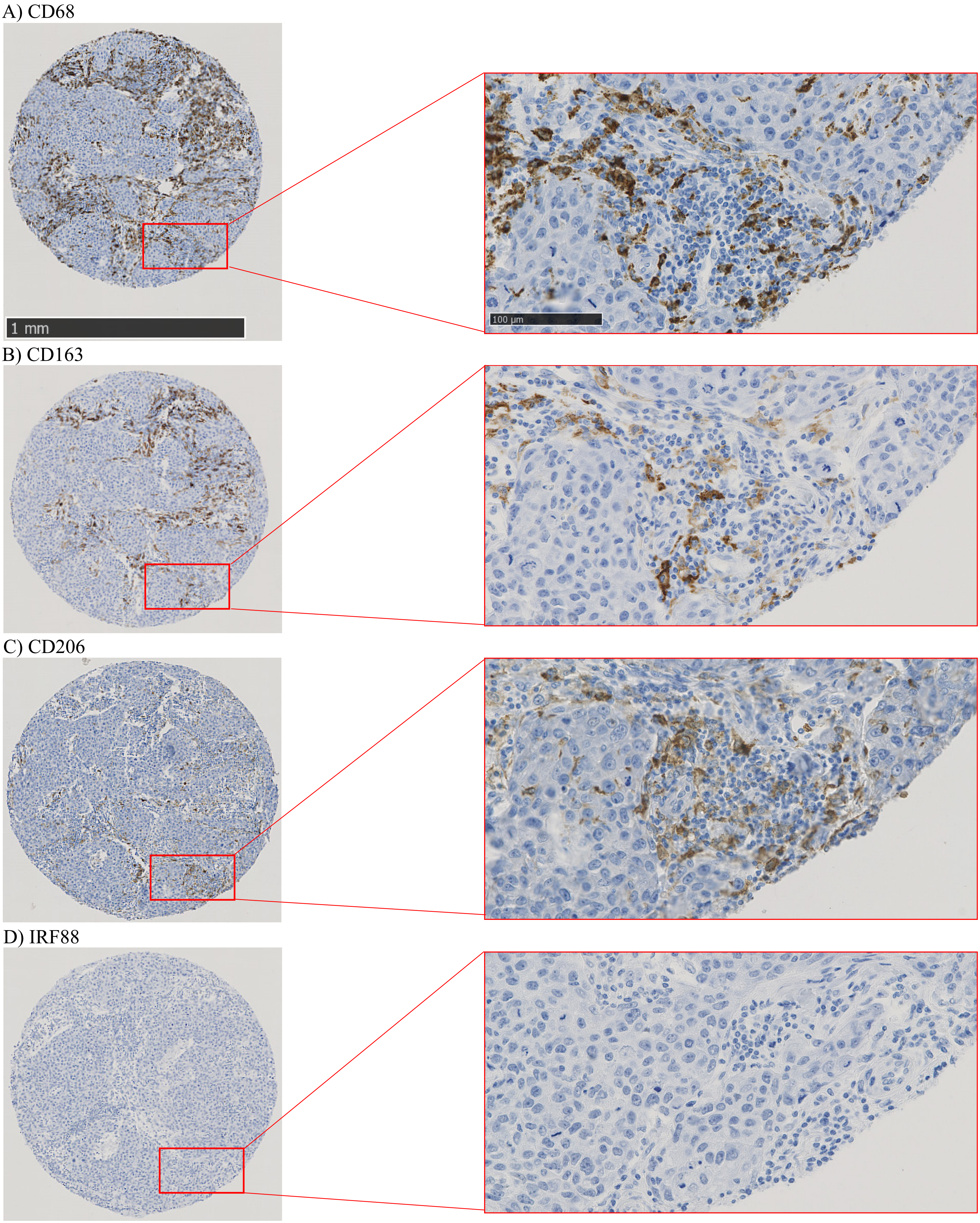

Supplementary figure S1: Examples of CD68 (A), CD163 (B), CD206 (C) and IRF8 (D) immunostaining in serial sections of a single tumor from patient 1, with x 40 magnification of a selected area (right side images).
