# Supplementary material for: Association of CD206 Protein Expression with Immune Infiltration and Prognosis in Patients with Triple-Negative Breast Cancer
Source: Cancers (Basel). 2022 Oct 3;14(19):4829. doi: 10.3390/cancers14194829 (PMC9564167; doi:10.3390/cancers14194829)
Supplement: Supplementary file 1 [file cancers-14-04829-s001.zip › Manuscript MacrophagesTNBC-Bobrie-SuppFigureS3.pdf]

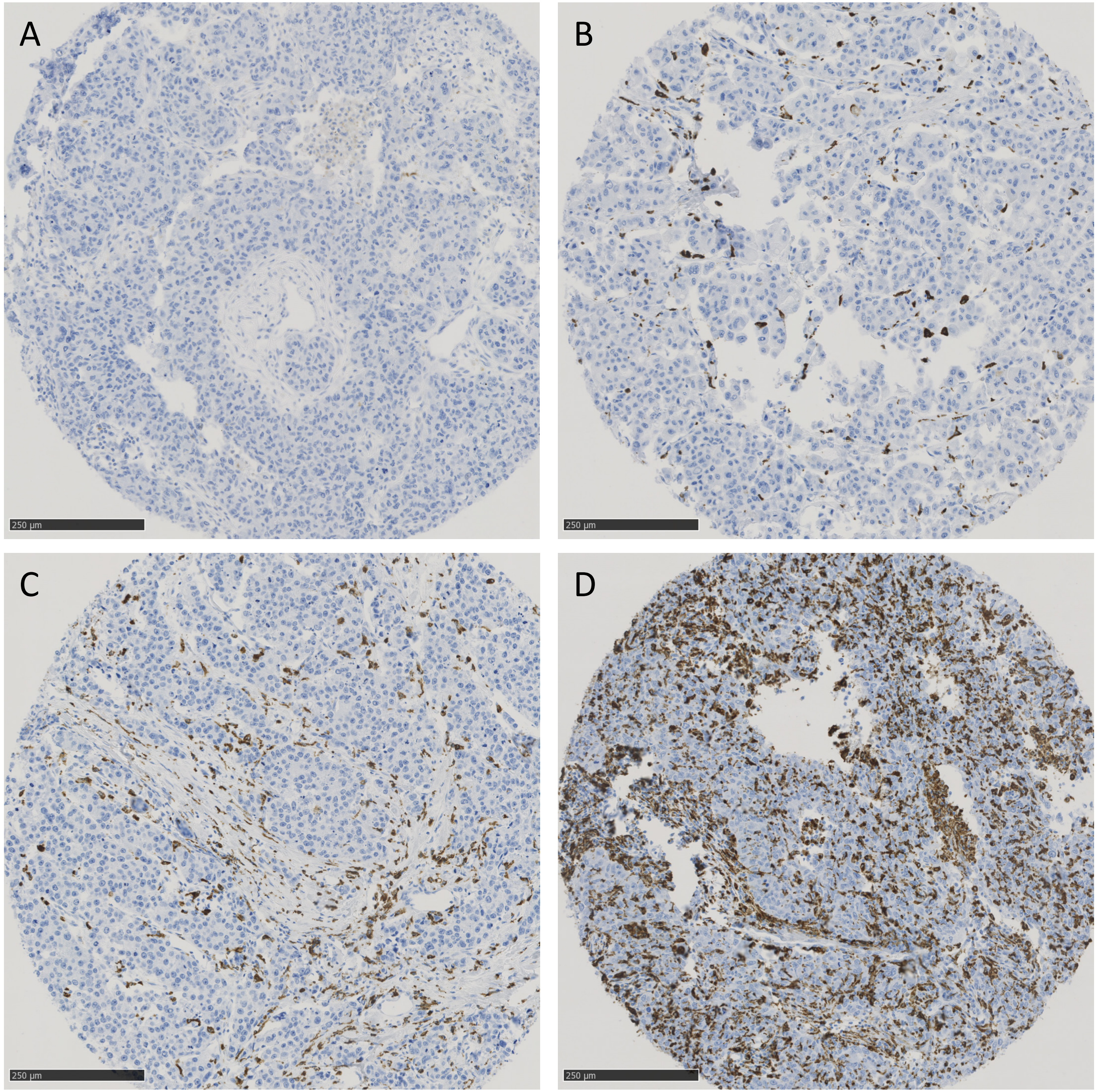

Supplementary figure S3: Quantification of CD68 expression by IHC. CD68^{+} macrophage infiltration was quantified as: absent/very low (score 0; A), weak (score 1; B), moderate (score 2; C), or strong (score 3; D). Scale bar: 250 µm.
