# Supplementary material for: Association of CD206 Protein Expression with Immune Infiltration and Prognosis in Patients with Triple-Negative Breast Cancer
Source: Cancers (Basel). 2022 Oct 3;14(19):4829. doi: 10.3390/cancers14194829 (PMC9564167; doi:10.3390/cancers14194829)
Supplement: Supplementary file 1 [file cancers-14-04829-s001.zip › Manuscript MacrophagesTNBC-Bobrie-SuppFigureS5.pdf]

CD206 prognostic significance in T1 tumors

A) OS B) RFS

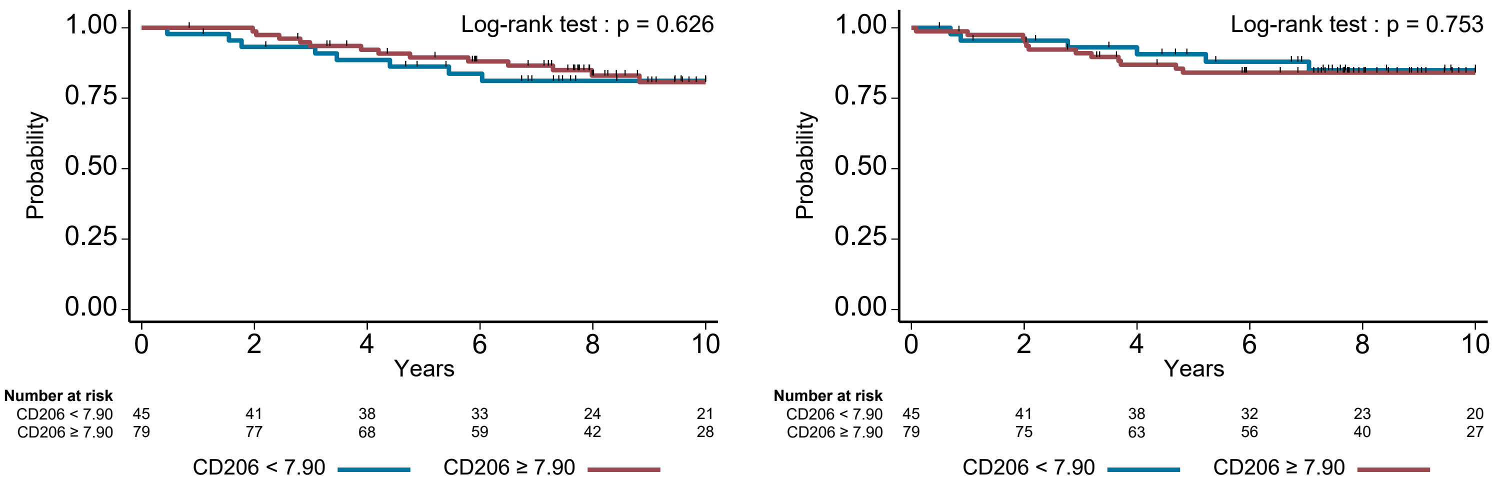

CD206 prognostic significance in ≥ T2 tumors

C) OS D) RFS

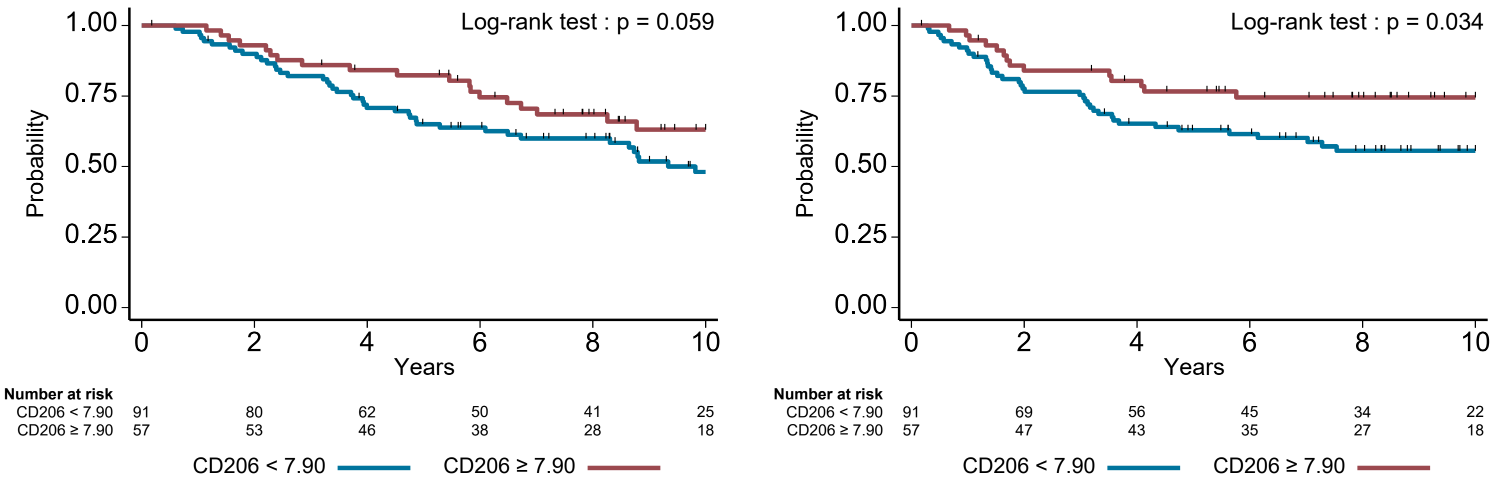

Supplementary figure S5: **Impact of CD206 expression on overall survival (OS) (A and C) and relapse free survival (RFS) (B and D) according to tumor size T1 (A and B) and tumor size ≥ T2 (C and D) expression levels.** Red lines represent high CD206 expression (≥7.90 cells/cm²) and blue lines represent low CD206 expression (< 7.90 cells/mm²). N=124 samples for T1 tumors ; N=148 samples for tumors ≥ T2.
