# Supplementary material for: Association of CD206 Protein Expression with Immune Infiltration and Prognosis in Patients with Triple-Negative Breast Cancer
Source: Cancers (Basel). 2022 Oct 3;14(19):4829. doi: 10.3390/cancers14194829 (PMC9564167; doi:10.3390/cancers14194829)
Supplement: Supplementary file 1 [file cancers-14-04829-s001.zip › Manuscript MacrophagesTNBC-Bobrie-Supplementary tables.pdf]

Macrophage marker	Number of patients (N = 285)	%
**CD68** (18 missing values)		
Low (Score 0 to 2)	173	64.79
High (Score > 2)	94	35.21
**CD163** (9 missing values)		
Low (Score 0 to 2)	200	72.46
High (Score > 2)	76	27.54
**CD206** median [min-max] (13 missing values)	7.90	[0 – 441.19]
Low (< 7.90 cells/mm²)	136	50.00
High (≥ 7.90 cells/mm²)	136	50.00
**IRF8** median [min-max] (8 missing values)	3.11	[0 – 387.06]
Low (< 3.11 cells/mm²)	139	50.18
High (≥ 3.11 cells/mm²)	138	49.82

Supplementary Table S1: TAM markers expression in TNBCs.

For each marker, low and high expression groups were defined. A semi-quantitative analysis of CD68 and CD163 expression was performed (scores 0 to 3) and the mean of duplicates was calculated for each patient. Low expression corresponds to scores 0 to 2, and high expression to scores >2. The total number of CD206- and IRF8-expressing TAMs was counted, and their densities reported as the number of positive cells per mm². The mean of duplicates was calculated for each patient, and the median density in the population was used to classify samples in low and high expression.

		CD68					IRF8					CD163					CD206				
		Low		High		*p*-value	Low		High		*p*-value	Low		High		*p*-value	Low		High		*p*-value
		N	%	N	%		N	%	N	%		N	%	N	%		N	%	N	%	
**CD68**																					
	Low						107	81.68	61	46.92	**<0.001**	156	81.25	15	20.55	**<0.001**	96	71.64	73	57.03	**0.013**
	High						24	18.32	69	53.08		36	18.75	58	79.45		38	28.36	55	42.97	
**IRF8**																					
	Low	107	63.69	24	25.81	**<0.001**						119	60.71	17	22.67	**<0.001**	119	60.71	17	22.67	**<0.001**
	High	61	36.31	69	74.19							77	39.29	58	77.33		77	39.29	58	77.33	
**CD163**																					
	Low	156	91.23	36	38.30	**<0.001**	119	87.50	77	57.04	**<0.001**						106	77.94	87	65.91	**0.028**
	High	15	8.77	58	61.70		17	12.50	58	42.96							30	22.06	45	34.09	
**CD206**																					
	Low	96	56.80	38	40.86	**0.013**	91	69.47	43	32.09	**<0.001**	106	54.92	30	40.00	**0.028**					
	High	73	43.20	55	59.14		40	30.53	91	67.91		87	45.08	45	60.00						

Supplementary Table S2: Correlation of CD68, IRF8, CD163 and CD206 expression levels.
